# Import of extracellular ATP in yeast and man modulates AMPK and TORC1 signalling

**DOI:** 10.1242/jcs.223925

**Published:** 2019-04-03

**Authors:** Gabriella M. Forte, Elizabeth Davie, Shervi Lie, Mirita Franz-Wachtel, Ashley J. Ovens, Tingting Wang, Jonathan S. Oakhill, Boris Maček, Iain M. Hagan, Janni Petersen

**Affiliations:** 1 Faculty of Biology, Medicine and Health, University of Manchester, Oxford Road, Manchester, M13 9PT, United Kingdom; 2 Flinders Centre for Innovation in Cancer, College of Medicine & Public health, Flinders University, Adelaide, SA 5001, Australia; 3 South Australia Health and Medical Research Institute, North Terrace, PO Box 11060, Adelaide SA 5000 Australia; 4 Proteome Center Tuebingen, University of Tuebingen, Auf der Morgenstelle 15, 72076 Tuebingen, Germany; 5 Metabolic Signalling Laboratory, St Vincent's Institute of Medical Research, School of Medicine, University of Melbourne, Victoria 3065, Australia; 6 Mary MacKillop Institute for Health Research, Australian Catholic University, Victoria 3000, Australia; 7 Cancer Research UK Manchester institute, Alderley Park, Macclesfield SK10 4TG, United Kingdom

**Keywords:** ATP, AMP, TORC1, AMPK, Ssp2, Tsc1, Tsc2, *Schizosaccharomyces pombe*, Fission yeast, Nutrient stress

## Abstract

AMP-activated kinase (AMPK) and target of rapamycin (TOR) signalling coordinate cell growth, proliferation, metabolism and cell survival with the nutrient environment of cells. The poor vasculature and nutritional stress experienced by cells in solid tumours raises the question: how do they assimilate sufficient nutrients to survive? Here, we show that human and fission yeast cells import ATP and AMP from their external environment to regulate AMPK and TOR signalling. Exposure of fission yeast (*Schizosaccharomyces pombe*) and human cells to external AMP impeded cell growth; however, in yeast this restraining impact required AMPK. In contrast, external ATP rescued the growth defect of yeast mutants with reduced TORC1 signalling; furthermore, exogenous ATP transiently enhanced TORC1 signalling in both yeast and human cell lines. Addition of the PANX1 channel inhibitor probenecid blocked ATP import into human cell lines suggesting that this channel may be responsible for both ATP release and uptake in mammals. In light of these findings, it is possible that the higher extracellular ATP concentration reported in solid tumours is both scavenged and recognized as an additional energy source beneficial for cell growth.

## Abstract (precis)

**Summary:** ATP and AMP import from external environments modifies growth control in yeast and man.

## INTRODUCTION

ATP is the key source of cellular energy storage in cells; however, ATP also plays a vital role as a signalling molecule. Extracellular ATP signalling regulates several biological processes including neurotransmission, pain sensation, cell differentiation, proliferation, cell death and the immune system (Bian et al., 2013; Dahl, 2015). ATP is released into the external environment by exocytosis, PANX1 channel-assisted export or through cell lysis following apoptosis (Dahl, 2015). Most of the signalling roles of ATP are mediated by the P2X7 receptor. The P2X7 receptor is an ATP-gated channel that, when activated, is permeable to Na^+^, Ca^2+^ and K^+^ ions to promote internal signalling. *In vitro* studies have shown that a high concentration of external ATP (5–10 mM) is toxic to cells. Import of adenosine generated from ATP breakdown accounted for this toxicity (Mello et al., 2014). In contrast, lower ATP levels (100 µM) promoted growth of mammalian cells (Erlinge et al., 1993; Van Daele et al., 1992). A real-time *in vivo* imaging study of ATP levels using a luciferase reporter, recorded external ATP concentrations in the micromolar range within the tumour microenvironment of HEK293T cells, while ATP was below detection levels in neighbouring normal tissues (Pellegatti et al., 2008).

Cells of solid tumours are frequently nutritionally stressed due to poor angiogenesis. The stressed nature of this existence raises the question as to whether environmental ATP may provide an additional energy source beneficial for growth of these stressed cancer cells and the associated host cells within the tumour. Early studies provided indirect evidence to suggest that extracellular ATP enters cells to increase intracellular adenine nucleotide concentrations (Chaudry, 1982). However ATP breakdown, adenosine uptake and internal ATP synthesis could not be excluded as routes to account for the elevation of internal ATP levels in these experiments.

The full conservation of growth controls and the ability to freely manipulate the environment of the single-celled fission yeast (*Schizosaccharomyces pombe*) make it an ideal system in which to study growth control and its coupling to cell fates including cell division and differentiation. Glutamate is a good nitrogen source for fission yeast cells as it is readily converted into glutamine, to provide a ready supply of nitrogen. In contrast, proline is considered to be a poor nitrogen source, as it must first be converted into glutamate before it can then be used for amino acid catabolism. A change in nitrogen source from glutamate to proline (nitrogen stress) inhibits target of rapamycin complex 1 (TORC1) activity (Hartmuth and Petersen, 2009). This TORC1 inhibition in response to nitrogen stress is driven by activation of Ssp2, the fission yeast energy-sensing AMPKα subunit (herein denoted AMPKα^Ssp2^) (Davie et al., 2015). AMPK is an energy sensor that couples eukaryotic cellular responses with changes in AMP:ATP ratios (Hardie, 2014). An increase in intracellular ATP levels reduces AMPK activity to increase TORC1 activity. In fission yeast, nitrogen stress is accompanied by a drop in ATP levels, AMPK activation and TORC1 inhibition (Davie et al., 2015). At 5 min after exposure to nitrogen stress, ATP levels were reduced but fully recovered to starting levels after another 5 min later. Thus, nitrogen stress induces a rapid and transient drop in cellular energy levels (Davie et al., 2015). This link between reduced cellular ATP levels, the ATP-responsive AMPKα^Ssp2^ and TORC1 control prompted us to ask whether the provision of external ATP to nitrogen-stressed cultures would provide an additional source of energy to reverse the cellular response to nitrogen stress and whether the extrapolation of any findings in yeast may be reiterated in human cells?

We find that both fission yeast and human cells can indeed import ATP from their external environment. The uptake of ATP modulates AMPK and TOR signalling networks upon addition to the growth media of both yeast and human cell lines. Exposure of fission yeast cells to AMP reduced the growth of wild-type cells, but had limited impact upon cells from which genes encoding AMPK had been deleted. Taken together, our observations show that external ATP can be scavenged and recognized in cells as an energy source. Thus, the abundant extracellular ATP found in solid tumours is likely to act as an additional energy source for the nutritionally stressed transformed cells and the associated host cells within these tumours.

## RESULTS

### External ATP blocks nitrogen stress-induced advancement of cell division

**Fig. 1.**
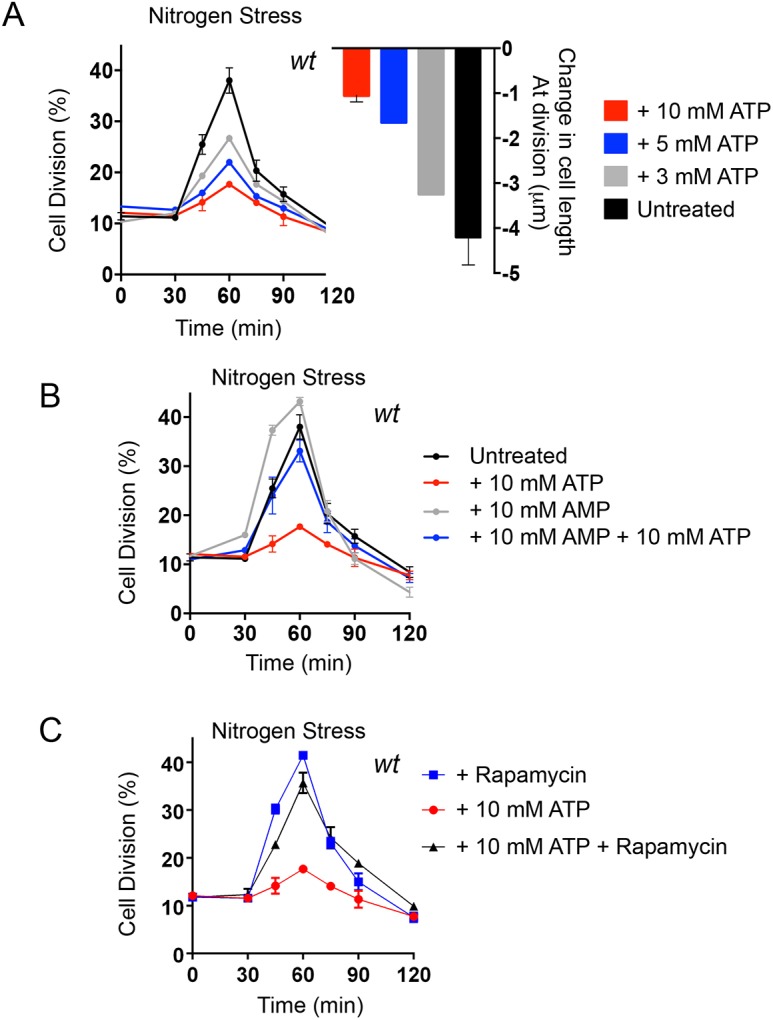
**ATP blocks the nitrogen-stress-induced advancement of mitotic onset.** (A) Early exponential prototroph wild-type (*wt*) cells, grown in EMMG, were filtered into EMMP containing either water (untreated) or the indicated concentrations of ATP. Samples were taken at the indicated time points to calculate the proportion of dividing cells (*n*=200 cells) and also measure cell length at division (*n*=100 cells of 0 and 120 min only). The left-hand graph shows the mean±s.e.m. proportion of dividing cells (%); the right-hand graph shows the mean±s.e.m. change in cell length at division after 120 min in the indicated media (μm). *n*=3. (B) Early exponential prototroph *wt* cells, grown in EMMG, were filtered into EMMP to induce nitrogen stress, containing 10 mM ATP, 10 mM AMP or an equal ratio of both (10 mM each). Samples were taken at the indicated time points to calculate the proportion of dividing cells. The graph shows the mean±s.e.m. proportion of dividing cells (%). *n*=3 (C) Early exponential prototroph *wt* cells, grown in EMMG, were collected by filtration, washed and re-suspended and filtered into EMMP to induce nitrogen stress, with the addition of either 10 mM ATP or 10 mM ATP+300 ng/ml Rapamycin. The graph shows the mean±s.e.m. proportion of dividing cells (%). *n*=3. Samples were taken at the indicated time points to calculate the proportion of dividing cells. In all panels, the same nitrogen-stressed cells with the addition of 10 mM ATP are shown.

Fission yeast cells respond to nitrogen stress cells by advancing mitotic onset to reduce the size at which cells divide. This response can be monitored as a sharp, but transient, rise in the frequency of dividing cells and a corresponding drop in cell size throughout the population (Fantes and Nurse, 1977; Petersen and Nurse, 2007). Because fission yeast grow by linear extension to generate a rod-shaped cell, the decline in cell size at division is directly reflected as a change in cell length at division. We therefore monitored the impact of exogenous ATP addition upon the nitrogen stress response by monitoring these changes in cell cycle progression. ATP was added to the medium at the same time as the nitrogen stress response was triggered by a switch from glutamate (good nitrogen source) into proline (poor nitrogen source) based medium. Cells were fixed at discrete intervals after the combined nitrogen stress and simultaneous addition of ATP. The fixed cells were stained with the cell wall stain Calcofluor to highlight the septa, which bisect dividing cells before constricting to split the parental cell into two daughters. By using a septum bisecting a non-constricting cell as a guide, we were able to restrict length measurements to dividing cells. Free cellular ATP levels in yeast have been estimated to be ∼1–2 mM (Ingram and Barnes, 2000; Özalp et al., 2010; Pluskal et al., 2011). Consistent with this, here we estimate, by means of liquid chromatography mass spectrometry (LC-MS), that free cellular ATP levels in EMMG-grown *S. pombe* cells are ∼2 mM (2.08±0.2 mM; mean±s.d.). We therefore began by providing similar external concentration of ATP through the addition of 3 mM ATP. We found that 3 mM ATP imposed a slight restraint on the advancement of mitotic onset that is always (Fantes and Nurse, 1977; Petersen and Nurse, 2007) invoked by this nutrient stress (Fig. 1A): both the peak in the frequency of dividing cells and the reduction in length at division were less pronounced than in untreated controls. Higher ATP concentrations accentuated the repression of the nitrogen stress response. Addition of 10 mM ATP at the time of shift more than halved the size of the peak of dividing cells seen in control cultures (17% versus 38%) (Fig. 1A) and cell length at division was reduced by only 1.05±0.15 μm as opposed to the 4.55±0.83 μm decrease in the controls.

The addition of 10 mM ATP to the EMMP growth medium changes the pH of the medium from 6.5 to 5.5. We therefore controlled for this pH shock through the inclusion of HCl in the EMMP into which cells were shifted to invoke the same degree of pH change and asked whether this pH shift alone had the same impact upon mitotic controls as ATP addition. Importantly, simultaneously shifting the pH of the proline medium from 6.5 to 5.5 at the initiation of nitrogen stress had no impact upon the advancement of cells into division (Fig. S1A). Thus, it is the elevation of external ATP levels, rather than the accompanying change in pH that blocks the rapid increase in the number of dividing cells ordinarily seen following nitrogen stress. This response supports the notion that cells sense external ATP as an increased energy source when nutritionally stressed.

### Changes in the external AMP:ATP ratio modulate nitrogen stress

In steady-state wild-type fission yeast cultures, ATP homeostasis is very efficiently maintained in different nutrient environments of decreasing quality, and the intercellular free AMP concentration is maintained at levels that are approximately half those of ATP (Pluskal et al., 2011; see also Fig. S1B). We previously showed that acute nitrogen stress activates AMPK and inhibits TORC1, that this response is transient and that ATP levels fully recover from a rapid drop to half the initial concentration within 10 min of invoking the stress (Davie et al., 2015). It is possible that the block to nitrogen-stress induced mitotic onset, imposed by the addition of exogenous ATP (Fig. 1), could arise through preventing the rapid and transient intracellular decrease in ATP:AMP ratios. If correct, this in turn would prevent the impact of nitrogen stress upon AMPK and, therefore, TOR signalling, and promote cell division (Davie et al., 2015; Petersen and Nurse, 2007). If this is the case, then simultaneous addition of equal concentrations of AMP and ATP to nitrogen-stressed cultures might be able to prevent the impact of ATP upon change of cell division kinetics. We therefore added equal concentrations of AMP and ATP to nitrogen-stressed cells at the same time of stress. Interestingly, simultaneous addition of AMP prevented the impact of exogenous ATP on the division kinetics (Fig. 1B). Moreover, the addition of AMP alone to nitrogen-stressed cultures accentuated the impact of the nitrogen stress to increase the proportion of cells dividing at the 60 min time point from 38% in controls to 43% in AMP-treated cells (Fig. 1B). Thus, exogenous AMP can invoke a low energy response upon nitrogen downshift and simultaneous addition of combined AMP and ATP can diminish the effect of ATP alone. These observations suggest that exogenous AMP and ATP have an instantaneous and brief impact on intracellular ATP:AMP ratios before ATP homeostasis is nevertheless naturally re-established (Davie et al., 2015; Pluskal et al., 2011). The opposing and rapid effects of extracellular AMP and ATP on the cellular response to nutrient stress makes it unlikely that our observations are due to breakdown of external nucleotides followed by the import of phosphates, which can then be used for synthesis of internal nucleotides. However, to test this possibility more directly, 10 mM Na_3_PO_4_ at pH 6.5 was added to nitrogen-stressed cells (Fig. S1C). The addition of free phosphate at the same concentration as ATP (Fig. 1) had no effect on the response of the cells to nitrogen stress, suggesting that the responses to external nucleotides that we observe here is due to ATP and AMP but not free PO_4_^3−^.

### Rapamycin blocks the impact of ATP upon nitrogen stress-accelerated division

The impact of ATP and AMP on nutrient-stressed cells (Fig. 1) suggest that exogenous nucleoside phosphates may be viewed as an enhanced energy supply by AMPK. As AMPKα^Ssp2^ sits above TORC1 in cell cycle controls (Davie et al., 2015), AMPKα^Ssp2^ inhibition (induced by elevation of ATP:AMP ratios) would prevent the transient inhibition of TORC1 that is normally induced by nitrogen stress. So a cell to which ATP was added would maintain lower AMPK activity and the extended cell size at division characteristic of growth in (good) glutamate medium, despite the switch to the poorer quality nitrogen source in proline medium. To test this hypothesis, we asked whether an alternative mode of TORC1 inhibition would overcome the ATP-dependent block on the advancement of mitotic commitment invoked by nitrogen stress. We have shown previously that addition of the TORC1 chemical inhibitor rapamycin to steady-state cultures grown in glutamate medium evokes a full nitrogen stress response; addition of rapamycin to cells grown in a good nitrogen source advances the timing of mitotic commitment and cell division even if these rapamycin-treated cells remain in a good nitrogen source (Petersen and Nurse, 2007). We therefore subjected wild-type cultures to nitrogen stress and simultaneously treated with either 10 mM ATP, rapamycin or a combination of both 10 mM ATP and rapamycin. A dramatic increase in dividing cells accompanied the combined addition of ATP and rapamycin during nitrogen stress (Fig. 1C). Thus, rapamycin treatment completely suppressed the impact of ATP on the control of cell division, leading us to conclude that exogenous ATP acts upstream of TORC1 to modulate the cell cycle control response to a change in nutrient quality.

### ATP alters nutrient stress invoked mitotic advancement via AMPK

**Fig. 2.**
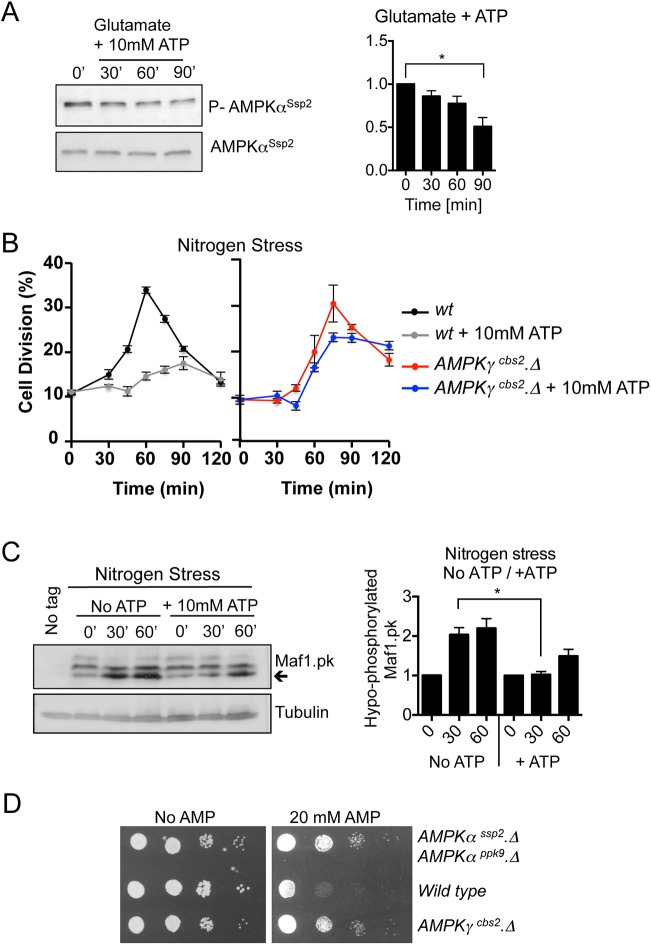
**ATP alters nutrient stress-invoked mitotic advancement via AMPK.** (A) Western blot analysis of AMPKα^Ssp2^ activating T-loop phosphorylation. Early exponential prototroph *wt* cells were grown in EMMG and 10 mM ATP was added. Samples were taken at the indicated time points (′ indicates minutes). *n*=3. (B) Early exponential prototroph *wt* and *AMPKγ^cbs2.^Δ* cells were grown in EMMG, and then filtered into EMMP to induce nitrogen stress, with and without the addition of 10 mM ATP. Samples were taken at the indicated time points to calculate the proportion of dividing cells. The graphs show the mean±s.e.m. proportion of dividing cells (%). *n*=3. (C) Western blot analysis of Maf1 phosphorylation. Early exponential prototroph *wt* cells, which have PK-tagged Maf1 (Du et al., 2012), were grown in EMMG and filtered into EMMP to induce nitrogen stress, with and without the addition of 10 mM ATP. Samples were taken at the indicated time points. Arrow highlights hypo-phosphorylated Maf1-PK. The quantification of the blots on the right shows mean±s.e.m. *n*=3. (D) Cell sensitivity growth assay. Early exponential prototrophic *wt*, *cbs2.Δ* and *ssp2.Δ ppk9.Δ* double-mutant cells, grown in EMMG. A 10-fold dilution series of each culture was spotted onto EMMG with or without 20 mM AMP. *P≤0.05 (two-tailed Student's *t*-test).

If external ATP alters intercellular ATP:AMP ratios, it would be expected to change AMPK activity. AMPKα^Ssp2^ phosphorylation of the T-loop by several kinases activates the AMPKα^Ssp2^ kinase; however, allosteric modulators, including AMP and glycogen, also regulate AMPK activity (Hardie, 2014). Interestingly, the addition of ATP to the growth medium of glutamate grown cells reduced T-loop phosphorylation of AMPKα^Ssp2^, which would be consistent with an elevation of ATP:AMP ratios (Fig. 2A) that reduces AMPK activity.

We have previously shown that AMPKα^Ssp2^ activation following nitrogen stress is regulated mainly by increased T-loop phosphorylation and that the regulatory γ-subunit of AMPK, to which AMP binds (Hardie, 2014), is not essential for the nitrogen stress response. This indicates that AMPKα^Ssp2^ is mainly activated independently of decreased ATP levels when controlling division in response to nitrogen stress.

We were therefore able to ask whether the γ-subunit of AMPK (fission yeast Cbs2; herein denoted AMPKγ^Cbs2^) was required for the modulation of the nitrogen stress response by external ATP. ATP did not block the advancement in mitosis in cells lacking AMPKγ^Cbs2^ (Fig. 2B). Thus, the AMPK γ-subunit is required to translate the exogenously supplied ATP into a change in cell cycle kinetics.

We next sought biochemical confirmation that exogenously supplied ATP blocks nitrogen-stress-induced inhibition of TORC1. Maf1, a repressor of RNA polymerase III, is a TORC1-specific substrate (Du et al., 2012; Michels, 2011). A collapse in the slower migrating phosphorylation forms, as the population of Maf1 shifts into the hypo-phosphorylated state, indicates that Maf1 phosphorylation (i.e. TORC1 activity) is reduced 30 min after the imposition of nitrogen stress (Davie et al., 2015). This inhibition of TORC1 recovers to pre-treatment levels within 90 min (Davie et al., 2015). Interestingly, the reduction in Maf1 phosphorylation was less pronounced when ATP was added to the growth medium at the time of nitrogen stress (Fig. 2C). Taken together, our data suggest that acute addition of ATP is sensed by the AMPK complex to maintain a lower AMPK activity and higher TORC1 activity, even though the AMPK complex should be responding to the nitrogen-induced stress that would normally enhance its activity (Davie et al., 2015).

We next assessed the impact of exposure to external AMP. Fission yeast strains were grown on glutamate medium to which AMP had been added. Growth of wild-type cells, but not AMPK mutants, was reduced by AMP (Fig. 2D), to suggest that AMPK-dependent sensing of AMP was responsible for this growth inhibition. This impact upon growth of wild-type cells indicates that AMPK senses AMP in the growth medium to modulate cell division controls when grown on a good nitrogen source.

### External ATP rescues the growth defect of Tsc1/2 mutants in fission yeast

**Fig. 3.**
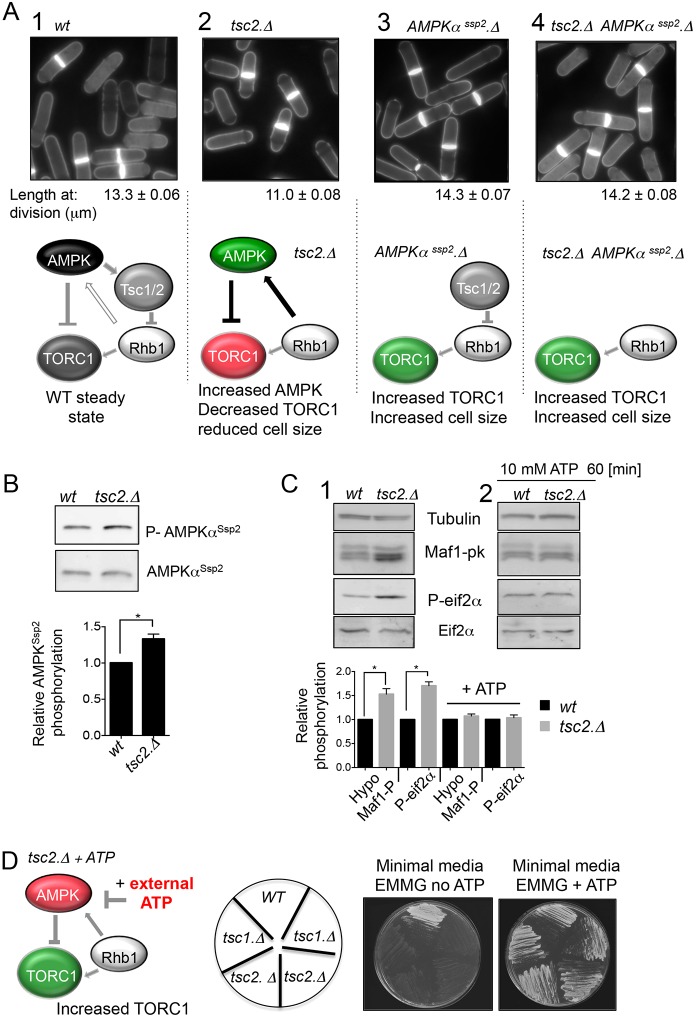
**ATP rescues the growth defect of *tsc1/2* mutants in fission yeast.** (A) Cell length at division of indicated strains for each strain for a minimum of 100 cells with complete septa were measured (mean±s.e.m.). Schematics illustrating: (1) that AMPKα^Ssp2^ regulates TORC1 though the fission yeast Tsc1/2 complex and Rhb1, as cells deleted for Tsc1/2 or mutant in *rhb1* are unable to downregulate TORC1 following nitrogen stress (Davie et al., 2015); (2) in *tsc2.Δ* mutants TORC1 activity is reduced presumably due to a gain-of-function ability in Rhb1 that leads to increased AMPK activity when the Tsc1/2 complex is absent, as has previously been reported in Tsc2^−/−^ MEFs (Lacher et al., 2010; Short et al., 2008); (3) in AMPKα^Ssp2^ deletion mutants TORC1 activity is enhanced, and (4) that deletion of AMPKα^Ssp2^ in *tsc2.Δ* mutants increases the cell length at division. (B) Western blot analysis and quantification (mean±s.e.m.; *n*=3) of AMPKα^Ssp2^ phosphorylation in *S. pombe* extracts from *wt* and *tsc2.Δ* cells grown in EMMG. (C) Western blot analysis and quantification (mean±s.e.m.; *n*=3) of Maf1 and Eif2α phosphorylation in *S. pombe* extracts from *wt* and *tsc2.Δ* cells grown in EMMG before and after the addition of 10 mM ATP. (D) Growth of *wt*, *tsc1.Δ* and *tsc2.Δ* cells (for *tsc1* and *tsc2* mutants JP823 and JP824 was used; see Table S1) on EMMG and EMMG with 20 mM ATP. *S. pombe* strains were grown exponentially in YES medium prior washing and re-suspension in EMMG. Then 2.5×10^5^ cells from each strain were streaked on EMMG with and without 20 mM ATP added, followed by incubation at 30°C for 48 h. Thus, addition of exogenous ATP phenocopies what is seen upon deletion of AMPKα^Ssp2^, and acts as an AMPK inhibitor to increase TORC1 activity and thus increase the cell size of *tsc2.Δ* mutants. *P≤0.05 (two-tailed Student's *t*-test).

We next asked whether external ATP could rescue the growth defect of fission yeast mutants with reduced TORC1 signalling. AMPK kinase modulates TORC1 activity via two routes. In the first, AMPK directly inhibits TORC1 through regulation of Raptor (Hardie, 2014). In the second, AMPK activates the tumour suppressor complex that comprises the tuberous sclerosis proteins, Tsc1 and Tsc2 (Tsc1 and Tsc2 function together as a complex are hereafter denoted Tsc1/2). Subsequently, Tsc1/2 inhibits the TORC1 activator Rheb (Garami et al., 2003; Inoki et al., 2003; Matsumoto et al., 2002). Consequently, loss of Tsc1/2 function in mammalian cells enhances TORC1 activity (Huang and Manning, 2008). We have previously shown that AMPKα^Ssp2^ regulates TORC1 though the fission yeast Tsc1/2 complex and Rhb1 (equivalent to human Rheb) (Fig. 3A1), as cells from which the genes encoding either component of the Tsc1/2 complex, or its target Rhb1 are deleted, are unable to downregulate TORC1 following nitrogen stress (Davie et al., 2015). This observation, along with reports from many other laboratories (reviewed in Aspuria et al., 2007), suggests that the regulation of TORC1 by Tsc1/2 and Rhb1 reported in mammals is conserved in wild-type fission yeast cells. It was therefore somewhat surprising when steady-state cultures of *tsc1/2* deletion mutants were reported to resemble rapamycin-treated cells in that they exhibited a reduction in cell size at division when grown on the minimal EMM2 medium that incorporates the optimal nitrogen source of ammonium (Weisman et al., 2007). We therefore also assessed the cell size at division of *tsc1/2* deletion mutants when grown in the EMMG medium used in this study. Consistent with the previous observations (Weisman et al., 2007), *tsc1* and *tsc2* deletion mutants also showed reduced cell size at division at the steady state when grown in EMMG (Fig. 3A1,2; Table S1). This reduction in size is reminiscent of the consequences of a constitutive reduction in TORC1 (Weisman, 2016). Interestingly, studies using Tsc2^−/−^ mouse embryonic fibroblasts (MEFs) reported that there was a Rheb-dependent feedback mechanism to increase AMPK activity when Tsc1/2 activity was lost; this Rheb control of AMPK was TORC1 independent (Lacher et al., 2010, 2011; Short et al., 2008). It is therefore likely that Rhb1 of fission yeast emulates this control to increase AMPKα^Ssp2^ activity in *tsc1*/*2*-deficient fission yeast cells and thus reduce the amplitude of TORC1 signalling through AMPK-dependent phosphorylation of the TORC1 component Raptor (fission yeast Mip1) (Fig. 3A2). The small enhancement of activating AMPKα^Ssp2^ phosphorylation in *tcs2* mutants (Fig. 3B) provides support for this hypothesis, as it suggests that AMPK activity may be elevated in the absence of the Tsc1/2 complex. To further address this hypothesis, we generated an *AMPKα^Ssp2^.Δ tsc2.Δ* double mutant, with the aim of blocking Rhb1 activation of AMPK while, at the same time, maintaining its essential function in the activation of TORC1 (Rhb1 function is essential; Mach et al., 2000). The introduction of the *AMPKα^Ssp2^.Δ* mutation into the *tsc2.Δ* background (*AMPKα^Ssp2^.Δ tsc2.Δ*) increased cell length at division beyond that of wild-type controls (Fig. 3A4, compare with that of the *AMPKα^Ssp2^.Δ* single mutation Fig. 3A3). This observation supports the notion that AMPK activity is increased in the *tsc2.Δ* mutant to reduce both TORC1 activity and therefore cell length at division. Decreased Maf1 phosphorylation in the *tcs2* deletion mutant confirms that removal of Tsc1/2 regulation in fission yeast reduces TORC1 activity in the minimal EMMG medium (Fig. 3C). Consistent with this, *tcs2* deletion mutants display increased Eif2α phosphorylation, a second consequence of TORC1 inhibition (Valbuena et al., 2012; Fig. 3C).

With the aim to assess whether external ATP could rescue the growth defect of mutants with reduced TORC1 signalling, we next exploited the ability of exogenously supplied ATP to inhibit AMPK (Fig. 2A). In *tsc2.Δ* cells exogenous ATP rescued the TORC1-dependent phosphorylation events to wild-type levels. Deletion of *tsc1* and *tsc2* from prototropic strains confers a growth deficiency on minimal medium (Fig. 3D) (van Slegtenhorst et al., 2004). The increase of AMPKα^Ssp2^ activity in cells lacking *tsc1*/*2* is likely to contribute to this growth defect. Because the exogenous provision of ATP reduces AMPK activity (Fig. 2C) and increases TORC1 signalling (Fig. 3C), we were now in a position to assess whether ATP could reverse the cell growth deficiency of *tsc1* and *tsc2* mutants. Importantly, the addition of exogenous ATP did indeed rescue all *tsc2.Δ* phenotypes; it returned cell size at division to wild-type lengths (Table S1), it rescued growth deficiency on minimal medium (Fig. 3D) and decreased TORC1 activity (Fig. 3C2).

In summary, both the Tsc1/2 complex and Rhb1 are required for environmental fine-tuning of TORC1 signalling (Aspuria et al., 2007); however, mutants lacking the Tsc1/2 complex have a reduced steady-state level of TORC1 signalling in minimal medium. Given the data from Tsc2^−/−^ MEFs (Lacher et al., 2010, 2011; Short et al., 2008) and our observation that the deletion of AMPK (*AMPKα^Ssp2^.Δ*) in the *tsc2.Δ* mutant background (*AMPKα^Ssp2^.Δ tsc2.Δ*) restored cell length at division to that of wild-type controls (Fig. 3A4), we assume that this decline in TORC1 activity arises from a gain of function of Rhb1 that increases AMPK activity when the Tsc1/2 complex is absent. Furthermore, key to this study, the addition of exogenous ATP reduced AMPK activity and rescued these multiple *tsc1/2* phenotypes, to provide further evidence for ATP uptake by cells.

### *S. pombe* AMPK is activated by AMP

**Fig. 4.**
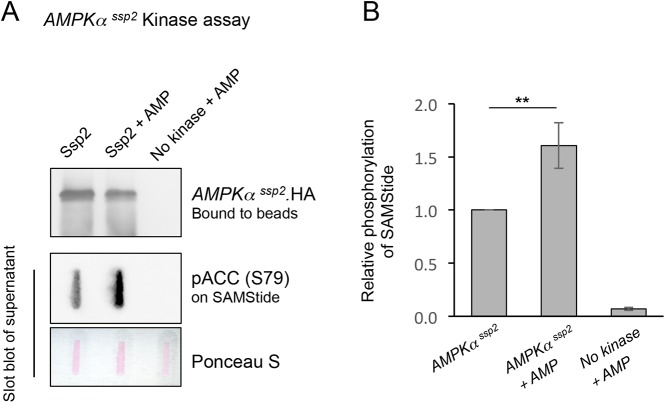
**The *S. pombe* AMPK Ssp2 is activated by AMP.** (A) Ssp2 immunoprecipitations followed by a kinase assay using SAMStide (a peptide containing the sequence around ACC S79 – an AMPK substrate) and pACC (S79) antibodies to detect Ssp2 activity. Following the enzyme reaction, the supernatant was used to perform a slot blot to detect pACC (S79) and the levels of Ssp2 kinase in reaction were determined by western blotting, 300 mM AMP was used. (B) Quantification of SAMStide phosphorylation under the conditions indicated. Results are mean±s.d., *n*=3. **P≤0.01 (two-tailed Student's *t*-test).

The data described above suggests that *S. pombe* AMPK is activated by an increase in AMP:ATP ratios. To look at this more directly, AMPK was immunoprecipitated from cells expressing HA-epitope tagged Ssp2 and used in a kinase assay with SAMStide as substrate [a peptide containing the sequence around acetyl-CoA-carboxylase (ACC) S79 – a known AMPK substrate] and an antibody against ACC phosphorylated at S79 [pACC (S79)] to detect AMPK activity. As predicted, the prior incubation of immunoprecipitated AMPK with AMP, promoted a 50% increase in kinase activity seen as increased phosphorylation of SAMStide (Fig. 4), indicating that, similar to the human AMPK complex, fission yeast AMPK is also activated by AMP. These finding suggests that *S. pombe* may be one of the simplest eukaryotes in which AMP directly regulates AMPK.

### Fission yeast cells import ATP from their external environments

**Fig. 5.**
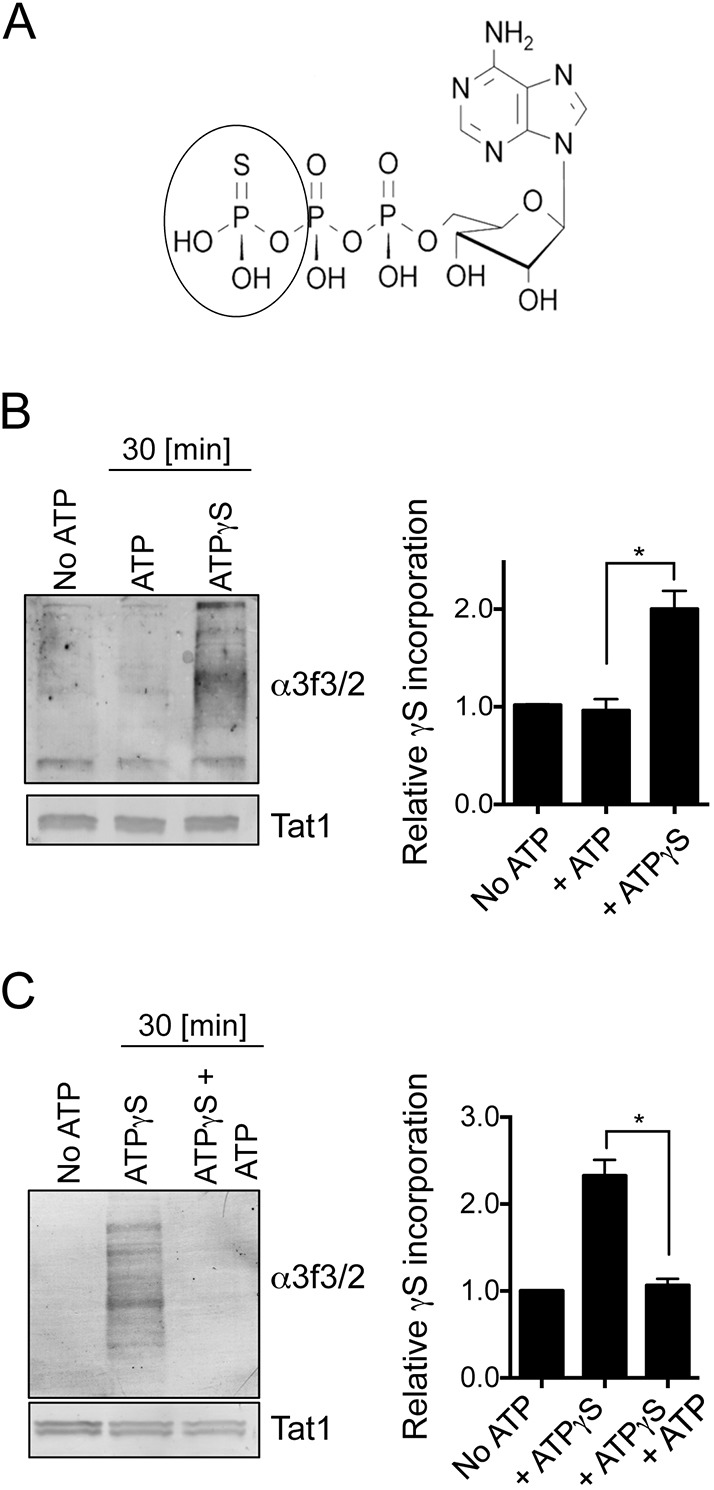
**Fission yeast import ATP from their external environments.** (A) Structure of ATP-γ-S. (B,C) Western blot analysis of ATP-γ-S import in *S. pombe*, and competition for import with ATP. Tat1 is shown as a loading control. Quantifications show mean±s.d.; *n*=3. *P≤0.05 (two-tailed Student's *t*-test).

To get further evidence for uptake of external ATP from the environment to be utilized by cells, we took advantage of the fact that the transfer of γ-thiol from ATP-γS by protein kinases to target proteins generates a bond that cannot be hydrolysed by phosphatases. Thus, the γ-thiol becomes irreversibly conjugated to substrates (Yasuoka et al., 1982) (Fig. 5A). A number of cellular kinases have affinity for ATP-γS (Cabail et al., 2016; Ikeda et al., 1991) and can accept it as an alternative to ATP from which to transfer the γ-thiol to endogenous target proteins. We therefore used the exogenous addition of ATP-γS to the medium to directly assess whether fission yeast assimilates environmental ATP.

The γ-thiol group that is transferred onto proteins from ATP-γS by protein kinases can be detected with either anti-3f3/2 antibodies (Cyert et al., 1988) or commercial anti-thiophosphate ester antibodies once *P*-nitrobenzyl mesylate (*p*-NBM) has been used to alkylate the thio-phosphorylation site (Allen et al., 2007). Fission yeast cells were nitrogen stressed as ATP-γS was added to the culture medium. Cells were harvested 30 min later and processed for western blotting using anti-3f3/2 antibodies in order to detect proteins onto which γ-thiol had been transferred by protein kinases. Addition of ATP-γS to growth medium promoted ‘thio-phosphorylation’ of fission yeast proteins (Fig. 5B), which supports the hypothesis that ATP enters the cells. Importantly, the uptake of ATP-γS could be out-competed by the addition of excess ATP (Fig. 5C), suggesting that the modes of ATP and ATP-γ-S uptake are similar.

### Human cell lines import ATP-γS from their environment

**Fig. 6.**
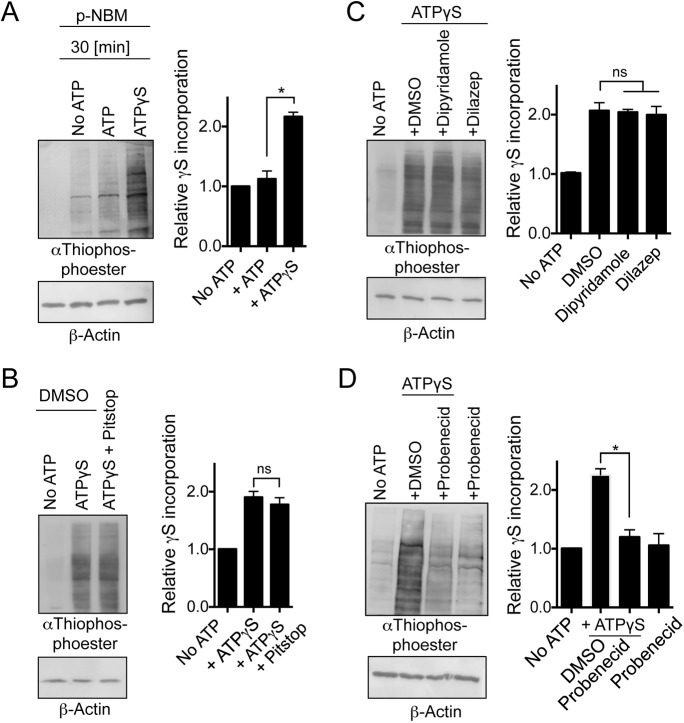
**Mammalian cells import ATP from their external environments to activate mTORC1.** (A) Western blot analysis of ATP-γ-S import in HEK293T cell extracts alkylated with *p*-NBM and probed with anti-thiophosphate ester. (B) Pitstop2 was used to inhibit endocytosis, (C) Dipyridamole and Dilazep to inhibit adenosine import and (D) probenecid to inhibit the organic anion transporter. Relative changes in phosphorylation compared to total protein levels are shown (mean±s.d.). *n*=3. β-actin is shown as a loading control. *P≤0.05; ns, not significant (two-tailed Student's *t*-test).

We next asked whether ATP-γS could also be imported into human HEK293T cells and used by endogenous kinases to thio-phosphorylate endogenous proteins. As with our observation in yeast (Fig. 5), thio-phosphorylation was observed in total protein extracts of HEK293T cells (Fig. 6A). To explore the mechanism by which ATP-γS was imported from the growth medium into HEK293T cells, we added Pitstop2 to block endocytosis (Dutta et al., 2012) and Dipyridamole and Dilazep to block adenosine import (Dawicki et al., 1985). Neither drug altered the pattern of thio-phosphorylation of endogenous proteins (Fig. 6B,C). The pannexin trans-membrane channel PANX1 regulates ATP release to the external environment in mammalian cells and is inhibited by probenecid (Silverman et al., 2008). Interestingly, probenecid addition to HEK293T cells blocked thio-phosphorylation of endogenous proteins (Fig. 6D), suggesting that this channel may be responsible for both release and uptake of ATP in mammalian cells.

**Fig. 7.**
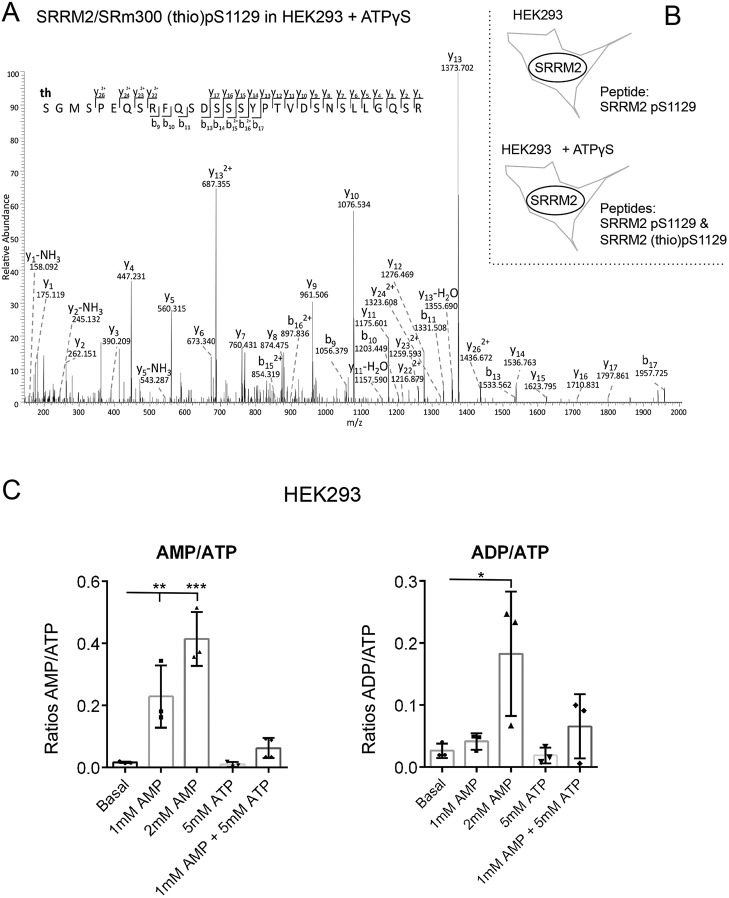
**Import of external nucleotides alters the internal nucleotide levels and promotes phosphorylation of nuclear protein.** (A) Fragmentation pattern of the SRRM2 thiophosphopeptide [(th)GMSPEQSRFQSDSSSYPTVDSNSLLGQSR] indicating S1129 is thio-phosphorylated. The mass of the parent ion is 1111.4728, the measured mass error is 0.0940 ppm. (B) The SRRM2 S1129 phosphopeptide was identified in both ATP-γ-S-treated and control samples (Fig. S1A,B), whereas thio-phosphorylated S1129 was only found when ATP-γ-S was added to the growth medium. (C) Adenine nucleotides and adenylate energy charge were measured by LC-MS from HEK293 perchlorate extracts. Results are mean±s.d. (*n*=3). *P≤0.05, **P≤0.01, ***P≤0.001 (one-way ANOVA).

Together, our results provide evidence for ATP uptake in yeast and mammalian cells as addition of exogenous ATP to the culture media resulted in: (1) a block to nitrogen stress-induced cell division; (2) modified AMPK and TORC1 signalling, (3) rescued cell growth of *tsc1/2* mutant cells; and (4) thio-phosphorylation in total protein extracts when ATP-γS was added to the culture medium. To gain further evidence for uptake from the environment, ATP-γS was added to the growth medium of HEK293 cells and total protein extract was examined by mass spectrometry. SRRM2 (also known as SRM300) is a nuclear protein involved in pre-mRNA splicing (Blencowe et al., 2000). We previously identified several phosphorylation sites including S1129 on SRRM2 (Franz-Wachtel et al., 2012). Here again, SRRM2 phosphorylated (p)S1129 was observed in protein extracts from both control and ATP-γS-treated cells; however, SRRM2 thio-pS1129 was only observed when ATP-γS was added to the HEK293 culture medium (Fig. 7A; Fig. S2) to consolidate the conclusion that ATP-γS is being actively taken up from the cellular environment. Finally, internal adenine nucleotide concentrations from HEK293 cells, to which nucleotides were added to the growth medium, were established. In agreement with the observation described above, measurements by LC-MS showed that the import of external nucleotides alters internal nucleotide pools to modulate the cells AMP:ATP and ADP:ATP ratios (Fig. 7C).

**Fig. 8.**
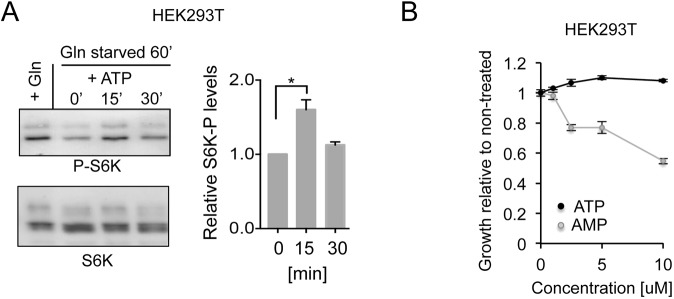
**External nucleotides regulate human mTORC1 and growth rates.** (A) Western blot analysis and quantification of AMPK and TORC1 substrate (S6K) phosphorylation in extracts from control HEK293T cells (+Gln) or HEK293T cells after transfer into the glutamate starvation medium for 60 min (Gln starved) and treatment with 2 mM ATP. Samples were taken at the indicated time point after ATP addition (′ indicates minutes). Relative changes compared to non-treated cells with standard deviation are shown in the graph, *n*=3. (B) Growth assay showing cell sensitivity to ATP and AMP. Average growth relative to non-treated along with standard deviation of *n*=3 experiments is shown. *P≤0.05 (two-tailed Student's *t*-test).

Acute ATP addition to the growth medium of glutamine-starved HEK293T cells promoted a quick and transient activation of mTORC1 (Fig. 8A), again indicating that cells take up ATP and that mTORC1 is activated. As with our observations in yeast (Fig. 2), this activation in cell cultures was transient. The addition of ATP had no impact on growth rate of non-starved HEK293T cells when compared to untreated controls. However, the addition of AMP reduced the growth of HEK293T cells to below that of controls (Fig. 8B).

Together, our data indicate that extracellular ATP and AMP are imported into both yeast and human cells, whereupon it is recognized as an energy source that regulates AMPK and TORC1 signalling and cell growth.

## DISCUSSION

Here, we describe how external ATP blocks nitrogen-stress-induced acceleration of mitosis and cell division in a dose-dependent manner. This influence of exogenous ATP upon cell cycle control suggests that changes in intracellular energy levels are being sensed. The reversal of this block to advancement of mitosis upon treatment with the TORC1 inhibitor rapamycin (Fig. 1C) suggests that this impact of ATP acts upstream of TORC1. ATP and AMP had opposing impacts upon the kinetics of the nitrogen-stress response. Moreover, combined treatment with ATP and AMP negated the effect of one another to support a wild-type response (Fig. 1B). ATP addition to yeast inhibited AMPK and transiently activated TORC1 (Fig. 2). Exposure to AMP reduced cell growth of wild-type fission yeast lines, but importantly not of AMPK-deficient fission yeast, suggesting that the block to cell growth imposed by elevated exogenous AMP levels is mediated by AMPK. Consistent with these observations, prior incubation of immunoprecipitated AMPK with AMP promoted a 50% increase in kinase activity (Fig. 4) indicating that, in contrast to what is seen in *S. cerevisiae* (Wilson et al., 1996), fission yeast AMPK, like its human counterpart, is activated by AMP. Together, these data support a model in which exogenous ATP and AMP regulate AMPK to modulate TORC1 and cell growth accordingly.

This impact of extracellular ATP and AMP upon intracellular growth controls, raises the question as to how these nucleoside phosphates alter the intracellular energy ratios that regulate AMPK activity. Upon addition of ATP in our physiological experiments, AMPK inhibition, TORC1 activation and a block to cell division were all observed within 30 min. This impact of the acute exposure to exogenous ATP would suggest that nucleotide uptake is rapid, a view supported by earlier, indirect, evidence that suggested extracellular ATP enters cells to increase intracellular adenine nucleotide concentrations (Chaudry, 1982). In steady-state growth, the intracellular level of free ATP in fission yeast cells is double that of free AMP, and ATP homeostasis is very efficiently maintained in different nutrient environments of decreasing nitrogen supply quality (Pluskal et al., 2011). Our nitrogen stress invoked an acute and transient halving of ATP levels; however, this decline was fully reversed within 10 min of the stress (Davie et al., 2015). This acute and transient reduction in ATP levels shows that intracellular ATP:AMP ratios are rapidly restored despite continuing exposures to stress. Thus, cells rapidly activate acute adaptation pathways to ensure that ATP:AMP ratios are maintained irrespective of changes in nitrogen supply. This ability of cells to efficiently regulate ATP homeostasis along with our observation that the impact of exogenous ATP on TORC1 activity is transient in both yeast and human cells, suggests that equilibria between intracellular and exogenous ATP concentrations is very unlikely to be established in liquid culture. The reason for this is unknown but may be due to instability of external nucleotides in this environment. In contrast, the rescue of cell growth on solid medium for *tsc1/2* double-mutants upon addition of ATP cells (Fig. 3) suggests that, in this environmental context, the impact of ATP is longer lasting. Again the reason for this is unclear, but it is possible that nucleotides are more stable in stationary environments, without mixing.

Interestingly, our data showed that simultaneous addition of AMP and ATP did not have the effect of either nucleoside alone. Clearly, if both nucleosides are imported into cells at the same rate, then the intracellular ratio of ATP:AMP would remain unchanged to cancel any impact that the elevation of either nucleoside phosphate would have when added alone, as seen in Fig. 1.

At present it is unclear how AMP enters cells and how yeast cells take up nucleoside phosphates, as no obvious ortholog of PANX1 is found in *S. pombe*. Future studies will address this. Addition of ATP-γS to growth medium promoted thio-phosphorylation of both fission yeast and HEK293T proteins (Figs 4B and 5A), which supports the hypothesis that ATP-γS enters the cells. Importantly, the uptake of ATP-γS could be out-competed by the addition of excess ATP (Fig. 4C). The addition of ATP had no impact on growth rate of HEK293T cells whereas the addition of AMP reduced the growth of HEK293T cells to below that of controls (Fig. 6); this indicates that it is not uptake of external adenosine, generated from ATP and AMP breakdown, that mediated the effect of these nucleotides on cell growth. The addition of Dipyridamole and Dilazep, to block adenosine import, also supports this view, as neither reduced the uptake or thio-phosphorylation of total protein extracts (Fig. 5). Furthermore, the identification of thio-phosphorylation of S1129 of the nuclear pre-mRNA splicing protein SRRM2 (Blencowe et al., 2000) provides further evidence for import of ATP.

Our data suggests that the mechanism by which extracellular ATP can enter mammalian cells is either directly via, or controlled by, channels in the plasma membrane. We show that ATP-γS uptake still occurs after blocking endocytosis and adenosine uptake; however, we were able to block import of external ATP-γS with the PANX1 channel inhibitor probenecid. As the PANX1 channel regulates ATP release to the external environment in mammalian cells (Silverman et al., 2008), the block to import upon PANX1 inhibition raises the possibility that this channel may be responsible for both release and uptake of ATP in mammalian cells and so play a vital role in nucleoside phosphate homeostasis in tissues and tumours. Importantly, bidirectional transport of ions and signalling molecules has previously been reported for connexon and pannexon channels (Cheung et al., 2014).

External ATP concentrations of ∼100–900 μM have been reported in the tumour microenvironment of HEK293T cells studied *in vivo* in mice (Pellegatti et al., 2008). These levels are similar to those that we added to the culture medium of HEK293T cells to have the marked impacts reported here. As solid tumours are frequently nutritionally stressed, due to their ineffective and underdeveloped vasculature, it may be possible that cells within the heart of a solid tumour increase their chances of survival by directly importing ATP to use as an alternative energy source. Whether cancer cells take up ATP from PANX1 channels in the *in vivo* setting is unclear at present. However, uptake of ATP may be possible in the tumour setting where extracellular ATP levels are high (Pellegatti et al., 2008), despite the fact that internal ATP concentrations are higher. Previous studies have demonstrated that increased external ATP levels blocks ATP release from cells in order to prevent ATP leakage from cells when external levels increase (Qiu et al., 2012). Therefore, under conditions of the high external ATP levels that we assess here, the PANX1 channel may revert to import of ATP. However, this is unclear at present. If the PANX1 channel is required for ATP uptake in *in vivo* settings, the inhibition of import via PANX1 channel blockade has clear and potentially significant therapeutic implications.

## MATERIALS AND METHODS

### *S. pombe* strains and growth conditions

Strains used in this study are listed in Table S2. Unless otherwise specified, cells were cultured at 28°C in Edinburgh minimal media (EMM2) (Fantes, 1977) using 20 mM L-glutamic acid (EMMG) or 5 g/l NH_4_Cl (EMM) as a nitrogen source (Petersen and Russell, 2016). Cells were grown exponentially for 48 h. To induce nitrogen stress, cells at early exponential phase of 1.5×10^6^ cells/ml were filtered, washed and re-suspended in EMM2 medium that had 20 mM L-proline as nitrogen source (EMMP). Cells were harvested by filtrations at the indicated time points to prepare protein extracts for western blotting or fixed for cell length and division ratio measurements. For stress/sensitivity growth assays, cells were grown in EMMG to a cell density of 1.5×10^6^ cells/ml. A 10-fold dilution series starting with 5×10^4^ cells was spotted onto the media indicated. For ATP treatment of *S. pombe*, cells were grown in EMMG to a cell density of 1.5×10^6^ cells/ml, washed and then re-suspended in EMMP containing either 10 mM ATP or 10 mM ATP-γ-S, or, for the competition assays, 10 mM ATP-γ-S and 30 mM ATP, before cells were harvested for western blotting 120 min later.

### Cell length and division ratio measurements

Yeast cells were fixed with 3% formaldehyde, washed with PBS and stained with 5 mM Calcofluor solution in PBS (cat. no. F3543, Sigma Aldrich). Dividing cells were counted (>200 cells counted per time point) and cell length at division was measured from >80 cells per sample. Images of cells were obtained using a CoolSNAP HQ2 CCD camera and processed with ImageJ.

### Western blotting

A TCA precipitation protocol was followed for *S. pombe* total protein extracts (Caspari et al., 2000). Mammalian cell protein extracts were prepared by washing cells with PBS followed by lysis with Laemmli buffer (62.5 mM Tris-HCl pH 6.8, 2% SDS, 10% glycerol, 0.01% Bromphenol Blue and 50 mM DTT), the extracts were then sonicated for 5 s. *p*-NBM-treated mammalian extracts were prepared by first washing cells in PBS, re-suspending in RIPA buffer (50 mM Tris-HCl pH 8150 mM NaCl, 1% NP40, 0.1% SDS, protease inhibitor cocktail) and mixing with an equal volume of reaction buffer (10 mM HEPES pH 7.5150 mM NaCl, 10 mM MgCl_2_). The extracts were then sonicated and treated with 2.5 mM *p*-NBM at 25°C for 1 h, after which Laemmli buffer was added.

The following dilutions of antibodies were used in this study: mouse anti-TAT1 (1:2000; kind gift from Keith Gull, Sir William Dunn School of Pathology, University of Oxford, UK), rabbit anti-pAMPK T172 to detect pSsp2 T189 (1:2000; cat. no. 07-681, Millipore UK Ltd.), rabbit anti-Ssp2 (1:500; raised by Eurogentec, Southampton; see Davie et al., 2015 for validation) mouse anti-PK tag (1:2000; cat. no. MCA2895GA, AbD Serotec), rabbit anti-peIF2α S51 (1:2000; cat. no. 07-760, Millipore), rabbit anti-eIF2α (1:500; cat. no. sc133132, Santa Cruz Biotechnology), rabbit anti-S6K (1:100; cat. no. 9202, Cell Signaling), rabbit anti-pS6K (T389) (1:500; cat. no. 9205, Cell Signaling), rabbit anti-thiophophate ester (1:5000, cat. no. ab92570, Abcam), rabbit anti-ACC (1:1000, cat. no. 3676, Cell Signaling) and anti-pACC S79 (1:1000, cat no. 3661, Cell Signaling), and mouse anti-β-actin (1:1000; cat. no. 8227, Abcam). Alkaline phosphatase-coupled secondary antibodies were used for all western blotting experiments followed by direct detection with NBT/BCIP (VWR) substrates on PVDF membranes.

### Ssp2 immunoprecipitation and kinase assay

3×10^8^ cells expressing HA-tagged Ssp2 were lysed at 4°C in a FastPrep^®^-24 homogeniser (MP Biomedicals) in 100 μl of immunoprecipitation (IP) buffer [50 mM Hepes pH 7.5, 100 mM KCl, 0.1 mM EDTA, 1 mM DTT, 0.2% Tween 20, 25 mM NaF, 2 mM Na_3_VO_4_, 25 mM sodium-β-glycerophosphate, 0.5 mM PMSF, 5 mM N-ethylmaleimide and EDTA-free protease inhibitor cocktail (Roche)]. A further 100 μl of IP buffer was added to the cell lysates and the lysates were cleared at 10,000 ***g*** for 10 min. The supernatant was added to protein A Dynalbeads (Invitrogen technologies) coupled to anti-HA antibodies (Santa Cruz Biotechnology; non-coupled were used as control) and incubated with rotation at 4°C for 1 h. The beads were then washed four times in IP buffer and twice in kinase assay buffer (KAB; 10 mM Hepes pH 7.5, 20 mM MgCl_2_ and 1 mM DTT). Beads were re-suspended in 30 µl KAB with or without 300 µM AMP made up in KAB and incubated for 10 min at 30°C. To start the kinase assay, a mix of 100 μM ATP and 150 μM SAMStide peptide (Millipore) made up in KAB was added to the washed beads and incubated at 30°C for 1 h. The supernatant was removed and subsequently heated to 80°C for 2.5 min and used to perform a slot blot on nitrocellulose membrane to detect phosphorylation of SAMStide using antibodies against p-ACC (S79) (Cell Signaling). The beads from this assay were re-suspended in sample buffer (100 mM Tris-HCl pH 6.8, 4% SDS, 0.2% Bromophenol Blue, 20% glycerol and 5 mM DTT) and heated to 70°C for 5 min to dissociate the immunoprecipitated protein prior to being subjected to SDS-PAGE followed by a western blot to detect Ssp2 with HA antibodies.

### Mammalian cell culture and treatments

Hep3B, HEK293 and HEK293T cells (ATCC) were grown and maintained in high-glucose (4.5 g/l) Dulbecco's modified Eagle's medium (DMEM) supplemented with 10% fetal bovine serum (FBS) and penicillin/streptomycin (100 units and 0.1 mg per ml, respectively). Cells were incubated at 37°C with 5% CO_2_.

For Pitstop2 treatment, cells were starved of serum for 1 h prior to treatment. Then, 20 μM Pitstop2, was added to culture medium 5 min prior to the addition of 2 mM ATP-γ-S followed by an incubation for a further 20 min. Cells were then harvested (500 ***g*** for 3 min) for *p*-NBM treatment.

Inhibition of adenosine import was performed by treatment with 10 μM Dilazep or 10 μM Dipyridamole to the culture medium 40 min prior to the addition of 2 mM ATP-γ-S; cells were then incubated for a further 20 min and then harvested for *p*-NBM treatment. Probenecid was used to inhibit the organic anion transporter. Water-soluble probenecid was added at 2.5 mM to cell culture medium 10 min prior to the addition of 2 mM ATP-γ-S, cells were then incubated for a further 20 min and then harvested for *p*-NBM treatment.

The HEK293T growth assay with various concentrations of ATP or AMP was performed by seeding HEK293T cells were at 2000 cells per well in a six-well plate. The cells were allowed to adhere for at least 16 h prior to the addition of ATP or AMP at 1, 2.5, 5, 10 or 100 μM. The medium was changed along with ATP or AMP addition every 2 days. Experiments proceeded for a total of 8 days. Cells were fixed and stained in Crystal Violet (0.5% Crystal Violet, 4% formaldehyde made up in PBS) for 15 min, washed with water and allowed to dry. Then dye was solubilized with 1% SDS and absorbance measure at 540 nm.

### Nucleotide measurements

Wild-type *S. pombe* cells were cultured in EMMG. Cells were then filtered and washed with PBS. Cells were washed off the filter with 400 µl cold 0.5 M perchloric acid. Cells were lysed mechanically with glass beads at 4°C in a FastPrep^®^-24 homogenizer (MP Biomedicals). Lysates were clarified by centrifugation at 16,000 ***g*** for 3 min at 4°C. Clarified extracts were neutralized with 100 µl cold 2.3M KHCO_3_ and incubated on ice for 5 min. Samples were centrifuged at 16,000 ***g*** for 3 min at 4°C. Supernatants were collected and snap-frozen for MS analysis. The physiological ATP concentration in EMMG was calculated based on the assumption that fission yeast is a cylinder and the average cell length is 11 μM and diameter is 3.3 μM.

HEK293T mammalian cells were cultured in DMEM containing 10% fetal bovine serum and 1% penicillin/streptomycin at 37°C with 5% CO_2_. Cells were incubated with fresh DMEM for 3 h, before replacing with fresh DMEM containing phenformin and/or adenine nucleotides (adjusted to neutral pH) as indicated. Post-treatment, cells were washed with ice-cold PBS and harvested by rapid lysis using ice-cold perchloric acid as described previously (Scott et al., 2015). Adenine nucleotides and adenylate energy charge were measured by LC-MS from *S. pombe* and HEK293 perchlorate extracts, as described previously (Scott et al., 2015).

### Detection of thio-phosphate by LC-MS and MS data processing

HEK293T cells were grown and maintained in high-glucose (4.5 g/l) DMEM supplemented with 10% FBS, 8 mM glutamine and 1% penicillin/streptomycin. Cells were incubated at 37°C with 5% CO_2_. At 24 h after plating, cells were washed with Dulbecco's phosphate-buffered saline (DPBS) and fresh medium containing no FBS was added to starve the cells for 2 h. After 2 h, starvation medium was removed and fresh full medium with or without 2 mM ATP-γ-S was added and cells were incubated for 2 h before being collected and extracted for MS or western blotting.

Cells collected for MS were extracted using denaturation buffer (6 M urea, 2 M thiourea and 1% n-octyl glucoside). Extract was centrifuged at 16,000 ***g*** for 10 min at 4°C to remove insoluble materials. Supernatant was collected and precipitated using cold acetone overnight. Protein precipitate was resuspended in water with protease inhibitor cocktail and freeze dried for shipping.

Dried proteins were resolved in digestion buffer (6 M urea, 2 M thiourea, 10 mM Tris-HCl, pH 8.0) and digested in solution with trypsin as described previously (Borchert et al., 2010) without reduction of cysteine bonds and carbamidomethylation of cysteine residues. 10% of the resulting peptides were directly desalted with C_18_ StageTips (Rappsilber et al., 2007) and further analysed on an EASY-nLC 1200 coupled to an LTQ Orbitrap Elite mass spectrometer (both Thermo Scientific).

The rest of the peptide mixture was purified on Sep-Pak 18 cartridges (Waters) and subjected to phosphopeptide enrichment by TiO_2_ beads as described previously (Taumer et al., 2018) with minor modifications: After washing TiO_2_ beads in loading solution and incubating them with DHB [20 mg/ml 2,5-dihydroxybenzoic acid in 80% acetonitrile (ACN), 1% trifluoroacetic acid (TFA)], they were added to the sample in a peptide to bead ratio of 2:1 (mg/mg). Elution from the beads was performed two times with 100 µl of 5% ammonia hydroxide solution in 60% acetonitrile (pH>10.5) and once with 10 µl 1% formic acid in 80% acetonitrile. Fractions were subjected to TiO_2_ enrichment four times. LC-MS analyses were performed on an EASY-nLC 1200 coupled to a Q Exactive HF mass spectrometer (both Thermo Scientific).

Separations of the peptide mixtures from direct injection was performed as described previously (Franz-Wachtel et al., 2012) with slight modifications; peptides were injected onto the column in HPLC solvent A (0.1% formic acid) at a flow rate of 500 nl/min and subsequently eluted with an 127 min segmented gradient of 10, 33, 50 and 90% of HPLC solvent B (80% acetonitrile in 0.1% formic acid) at a flow rate of 200 nl/min. Precursor ions were acquired in the mass range from *m*/*z* 300–2000 in the Orbitrap mass analyser at a resolution of 120,000. The 15 most-intense ions were sequentially isolated and fragmented in the linear ion trap using collision-induced dissociation (CID) at a default CID settings. The target values for the MS scan and MS/MS fragmentation were 10^6^ and 10^5^ charges, with maximum fill times of 100 ms and 25 ms, respectively. Sequenced precursor masses were excluded from further selection for 60 s.

Enriched phosphopeptides were eluted with a 57-min segmented gradient of 10, 33, 50 and 90% of HPLC solvent B (80% acetonitrile in 0.1% formic acid). A full scan was acquired in the mass range from *m*/*z* 300 to 1650 at a resolution of 60,000 followed by HCD fragmentation of the seven most-intense precursor ions. High-resolution HCD MS/MS spectra were acquired with a resolution of 60,000. The target values for the MS scan and MS/MS fragmentation were 3×10^6^ and 10^5^ charges, with maximum fill times of 25 ms and 220 ms, respectively. Precursor ions were excluded from sequencing for 30 s after MS/MS.

MS data were processed using default parameters of the MaxQuant software (v1.5.2.8) (Cox and Mann, 2008). Extracted peak lists were submitted to database search using the Andromeda search engine (Cox et al., 2011) to query a target-decoy (Elias and Gygi, 2007) database of *H. sapiens* proteome (91,675 entries, downloaded on 23rd of October 2015) and 285 commonly observed contaminants.

In database search, full tryptic specificity was required and up to two missed cleavages were allowed. Protein N-terminal acetylation, oxidation of methionine, phosphorylation, and thiophosphorylation of serine, threonine, and tyrosine were set as variable modifications, whereas no fixed modification was defined. Initial precursor mass tolerance was set to 4.5 ppm, and 0.5 Da (CID) and 20 ppm (HCD) at the fragment ion level. Peptide, protein and modification site identifications were filtered at a false discovery rate (FDR) of 0.01.

### Statistical analysis

Data analyses were performed using GraphPad Prism 6 software. Statistical significance was calculated using two-tailed Student's *t*-test or a one-way ANOVA followed by a Duncan’s post-hoc to show significant difference between groups. *P*≤0.05 was considered statistically significant (*P≤0.05, **P≤0.01, ***P≤0.001, ****P≤0.0001).

## Supplementary Material

Supplementary information
