# Supplementary material for: Import of extracellular ATP in yeast and man modulates AMPK and TORC1 signalling
Source: J Cell Sci. 2019 Apr 3;132(7):jcs223925. doi: 10.1242/jcs.223925 (PMC6467490; doi:10.1242/jcs.223925)
Supplement: Supplementary information [file joces-132-223925-s1.pdf]

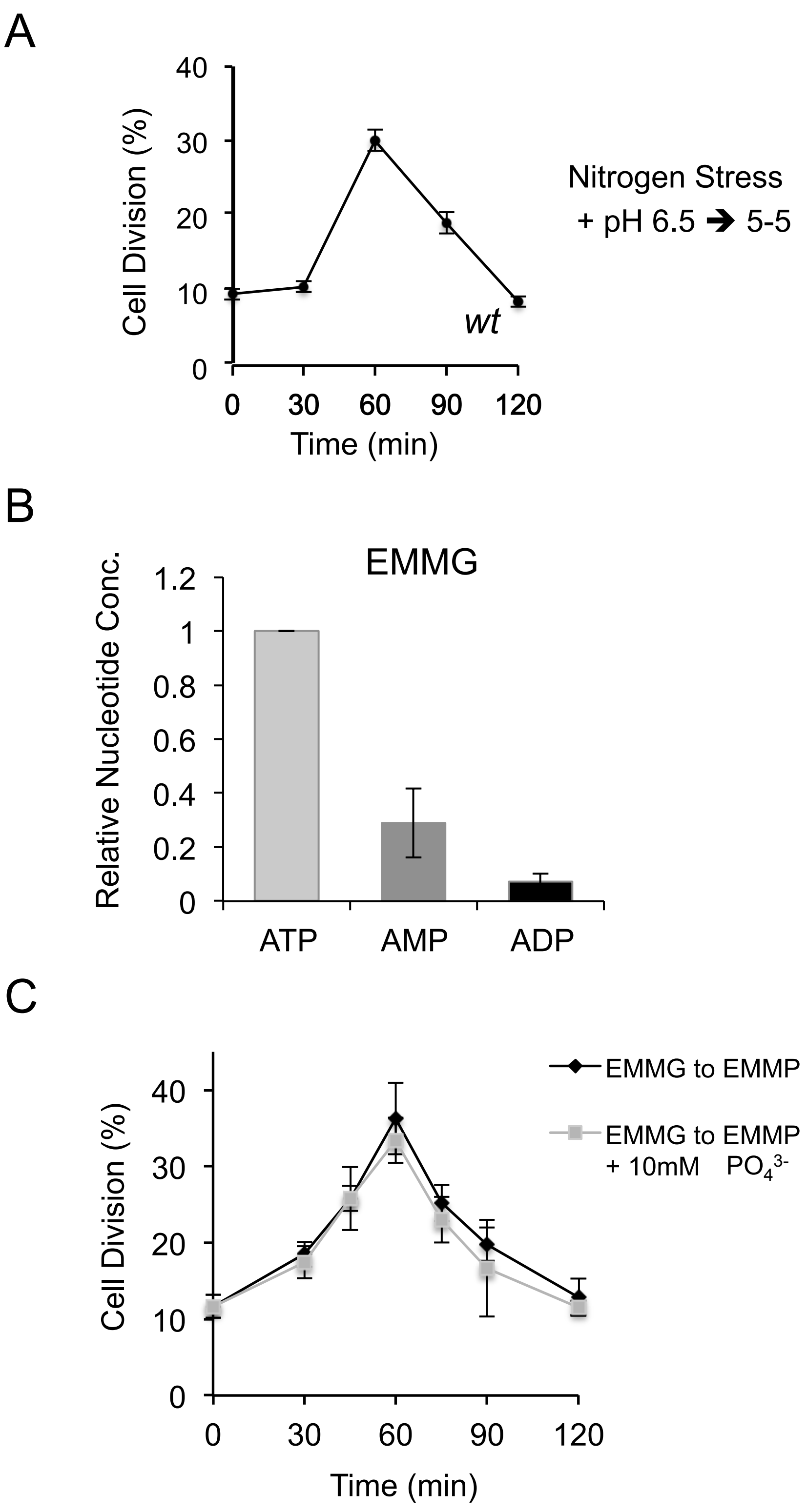

Figure S1. Import of phosphates or change in pH do not account for the effects of extracellular ATP on the cellular response to nutrient stress.

A) Early exponential prototroph *wt* cells, grown in EMMG, were filtered into EMMP which has had its pH adjusted to pH5.5 through the addition of HCl, to simulate the change in pH when ATP is added to media. Samples were taken to at the indicated time points to calculate the proportion of dividing cells. **B**) Measurements of nucleotide levels in cell grown in EMMG. **C)** Early exponential prototroph *wt* cells, grown in EMMG, were filtered into EMMP or to EMMP to which 10mM phosphate $P0_4^{3-}$ at pH 6.5 was added. Samples were taken to at the indicated time points to calculate the proportion of dividing cells.

Journal of Cell Science • Supplementary information

A

SRRM2/SRm300: pS1129 in HEK293T cells

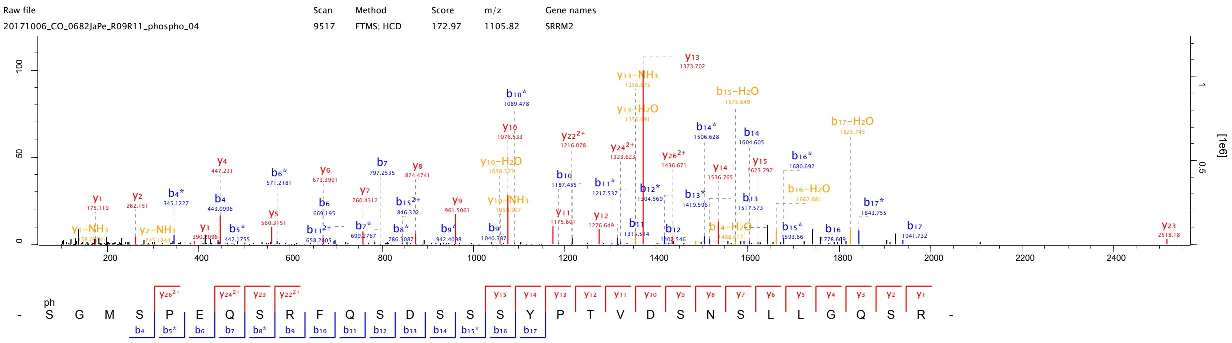

B

SRRM2/SRm300 pS1129 in HEK293T + ATPγS cells

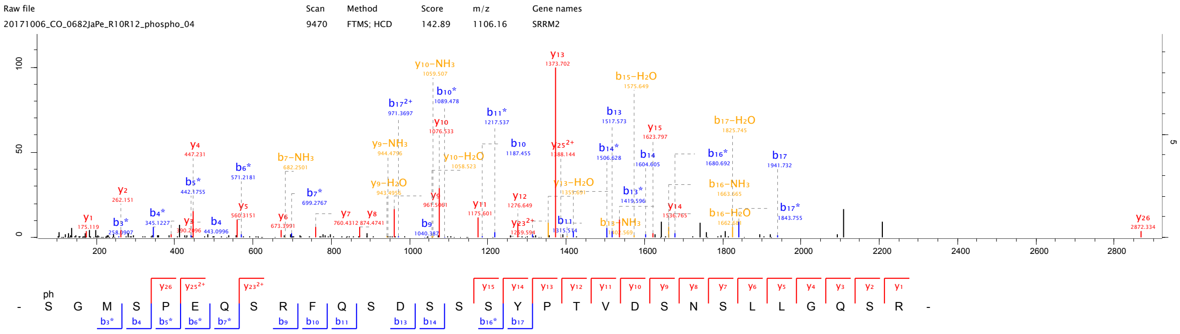

Figure S2. Detection of phosphorylated SRm300 pS1129 in HEK293T cells.

A,B) Fragmentation pattern of the SRRM2 phosphopeptide S(p)GMSPEQSRFQSDSSSYPTVDSNSLLGQSR indicating S1129 to be phosphorylated in HEK293T cells and HEK293T + ATPγS cells.

Journal of Cell Science • Supplementary information

Genotype	Cell length at division (μm)
wt	13.7 ± 0.06
tsc1.Δ	10.3 ± 0.08
tsc1.Δ + ATP	13.3 ± 0.08
tsc2.Δ	11.0 ± 0.08
tsc2.Δ + ATP	13.3 ± 0.1

Table S1: Cell length at division of indicated strains.

Cell were grown in EMMG +/- ATP and cell length at division +/- SD were measured, more than 100 cell were measured for each strain.

Table S2: . Yeast Strains used in this study

JP3	*h-*	lab stock
JP350	*h+*	lab stock
JP823	*h- tsc1::kanMx6*	E. Henske (van Slegtenhorst et al, 2004)
JP824	*h- tsc2::kanMx6*	E. Henske (van Slegtenhorst et al, 2004)
JP1079	*h- tsc2::kanMx6*	This study
JP1081	*h+ tsc1::kanMx6*	This study
JP1591	*cbs2::kanMx6*	From (Davie et al, 2015)
JP1624	*ssp2::ura4+ ppk9::ura4+*	From (Davie et al, 2015)
JP1731	*h- Maf1.pk:kanMx6*	From (Du et al, 2012)

Davie E, Forte GM, Petersen J (2015) Nitrogen regulates AMPK to control TORC1 signaling. *Curr Biol* **25:** 445-454

Du W, Hálová L, Kirkham S, Atkin J, Petersen J (2012) TORC2 and the AGC kinase Gad8 regulate phosphorylation of the ribosomal protein S6 in fission yeast. *Biology Open* **1:** 884-888

van Slegtenhorst M, Carr E, Stoyanova R, Kruger WD, Henske EP (2004) Tsc1+ and tsc2+ regulate arginine uptake and metabolism in Schizosaccharomyces pombe. *J Biol Chem* **279:** 12706-12713
